# Long-term impact and biological recovery in a deep-sea mining track

**DOI:** 10.1038/s41586-025-08921-3

**Published:** 2025-03-26

**Authors:** Daniel O. B. Jones, Maria Belen Arias, Loïc Van Audenhaege, Sabena Blackbird, Corie Boolukos, Guadalupe Bribiesca-Contreras, Jonathan T. Copley, Andrew Dale, Susan Evans, Bethany F. M. Fleming, Andrew R. Gates, Hannah Grant, Mark G. J. Hartl, Veerle A. I. Huvenne, Rachel M. Jeffreys, Pierre Josso, Lucas D. King, Erik Simon-Lledó, Tim Le Bas, Louisa Norman, Bryan O’Malley, Thomas Peacock, Tracy Shimmield, Eva C. D. Stewart, Andrew K. Sweetman, Catherine Wardell, Dmitry Aleynik, Adrian G. Glover

**Affiliations:** 1 National Oceanography Centre, European Way, Southampton, UK; 2 Natural History Museum, Cromwell Road, South Kensington, London, UK; 3 School of Environmental Sciences, University of Liverpool, Liverpool, UK; 4 Ocean and Earth Science, University of Southampton Waterfront Campus, European Way, Southampton, UK; 5 Scottish Association for Marine Science, Oban, Argyll, UK; 6 British Geological Survey, The Lyell Centre, Research Avenue South, Edinburgh, UK; 7 Heriot-Watt University, Riccarton, Edinburgh, UK; 8 Institut de Ciències del Mar (ICM-CSIC), Barcelona, Spain; 9 Eckerd College, Saint Petersburg, FL USA; 10 Massachusetts Institute of Technology, Cambridge, MA USA

**Keywords:** Biodiversity, Marine biology, Environmental impact

## Abstract

Deep-sea polymetallic nodule mining is in the exploration phase at present with some groups proposing a move towards extraction within years^1^. Management of this industry requires evidence of the long-term effects on deep-sea ecosystems^2^, but the ability of seafloor ecosystems to recover from impacts over decadal scales is poorly understood^3^. Here we show that, four decades after a test mining experiment that removed nodules, the biological impacts in many groups of organisms are persistent, although populations of several organisms, including sediment macrofauna, mobile deposit feeders and even large-sized sessile fauna, have begun to re-establish despite persistent physical changes at the seafloor. We also reveal that areas affected by plumes from this small-scale test have limited detectable residual sedimentation impacts with some biological assemblages similar in abundance compared to control areas after 44 years. Although some aspects of the modern collector design may cause reduced physical impact compared to this test mining experiment, our results show that mining impacts in the abyssal ocean will be persistent over at least decadal timeframes and communities will remain altered in directly disturbed areas, despite some recolonization. The long-term effects seen in our study provide critical data for effective management of mining activities, if they occur, including minimizing direct impacts and setting aside an effective network of protected areas^4,5^.

## Abstract (web summary)

Four decades after a test mining experiment that removed nodules, the biological impacts in many groups of organisms persist, although populations of several organisms have begun to re-establish despite persistent physical changes at the seafloor.

## Main

Recent rapid growth in exploration for polymetallic nodule deposits is raising societal awareness of deep-sea mining^6^. More than 21 billion tonnes of nodules, potato-sized mineral aggregations rich in critical metals such as cobalt and nickel, are estimated to lie on the abyssal seabed of the Clarion–Clipperton Zone (CCZ, North Pacific)^7^. However, Pacific nodule fields also sustain highly specialized animal and microbial communities with low abundance and biomass, but high species diversity compared to other deep-sea sedimented communities^8–11^ with most of the species still undescribed^12^. Falling beyond national jurisdiction, the seafloor mineral resources of the CCZ are regulated by the International Seabed Authority, which is at present developing the legal, financial and environmental framework to underpin any potential full commercial exploitation, if it occurs. Robust understanding of the effects of mining disturbance is thus urgently needed^13^.

The expected high sensitivity of abyssal communities to change combined with the potential spatial and temporal scales of mining operations (for example, roughly 400 km^2^ yr^−1^ of mining per operation with expected 20-year mine life^14,15^) sets them apart from most other anthropogenic stressors in the deep sea. Nodule mining is expected to cause immediate effects on the seabed surface and habitat in the path of collector vehicles, including mechanical disturbance, hard substratum habitat removal and sediment compaction. It will generate sediment plumes in the water column that can redeposit beyond mined areas^2^ causing biogeochemical alterations of the sediment and increased water turbidity at scales that could have significant impacts on ecosystems^3,16^. Recent estimates suggest plume redeposition could expand the visible seabed footprint several kilometres beyond the extent of test mining operations^17,18^. Over the multi-decadal life of a single operation, impacts from direct disturbance and plumes could extend over hundreds of square kilometres^19^ and cumulative impacts of many operations could be greater. However, biological effects of these physio-chemical alterations remain poorly understood, particularly over long timescales. Evaluation of the potential resilience of abyssal ecosystems to cumulative effects is largely constrained by the scarcity of full-scale experimental tests, and little is generally known about long-term recovery or succession patterns in abyssal ecosystems^4^. In this study, we define ‘recovery’ as a return to the original state of the ecosystem stated in terms of the parameter assessed, which includes a range of physical and biological characteristics, such as substratum composition and biological abundance. It does not indicate a full return of the ecosystem and its diversity to predisturbance conditions, which does not always occur in any environment^20^ and may be impossible with nodule removal^5^.

The most comprehensive recovery studies in the abyssal Pacific have been conducted outside the CCZ. This previous work has focused on the disturbance and recolonization experiment (DISCOL) in the Peru Basin, an area considerably less oligotrophic than the CCZ^21^ and at present not of commercial interest for mining. These studies showed persistent ecological impacts for some parameters 26 years after disturbance^22–24^ with some evidence of recovery in others^25^. The lack of information generally and specifically in the CCZ is a key evidence gap that is challenging the development of effective regulations for deep-sea mining that preserve biodiversity and ecosystem processes^26^. Access to longer-term recovery sites, such as those in the CCZ affected in the 1970s by seafloor collector tests^3^, is one of the few approaches available to help constrain the potential for recovery and timescales required.

Here we combine recently discovered archive material from the Ocean Minerals Company (OMCO) 1979 mining collector test (including location, engineering details and contemporaneous seafloor photographs) with a detailed field evaluation of the area from March 2023 aimed at assessing long-term environmental responses and recovery trajectories to nodule mining disturbance. These observations of aspects of ecological recovery 44 years after mining disturbance provide essential knowledge to inform conservation management strategies and decision-making for the future of deep-sea mining.

**Fig. 1:**
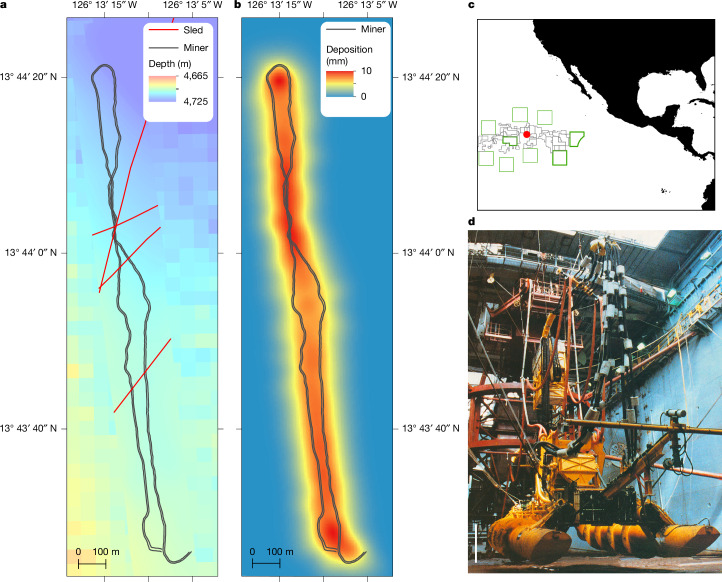
Maps of the OMCO track area. **a**, Multibeam bathymetry map overlaid with interpreted tracks of mining vehicle (in the photograph, **d**) and epibenthic sleds used for nodule collection in 1978. **b**, Modelled sediment deposition from plume generated by mining vehicle with central point of mining vehicle track overlaid (black). **c**, General location of test area (red dot) in the CCZ showing International Seabed Authority designated Areas of Particular Environmental Interest (green) and exploration contract areas (grey). Datum WGS 1984 for all maps. **d**, Photograph of a mining vehicle in the moon pool of the *Hughes Glomar Explorer* vessel in 1979. Scale bar, 2 m. Credit: Photo in **d** from ref. ^39^, reproduced under a Creative Commons licence CC BY 4.0.

**Extended Data Fig. 1:**
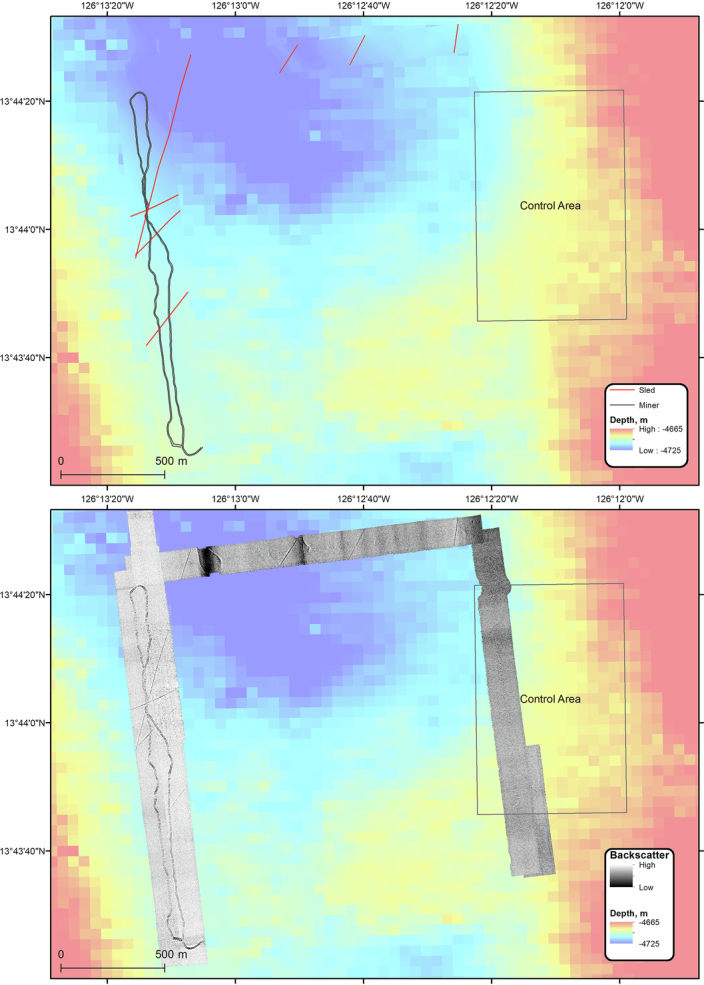
Map of study area showing tracks and control area. Top: ROV and shipboard multibeam bathymetry map with interpreted miner and epibenthic sled tracks. Bottom: Shipboard bathymetry map overlaid with multibeam backscatter from ROV survey, clearly showing the mining vehicle and epibenthic sled tracks. Position in degrees minutes seconds (WGS84).

The OMCO test (Fig. 1) set the blueprint for most modern CCZ operations: a roughly 10-m wide, self-propelled remotely operated mining vehicle connected by a riser pipe and pump system to a surface ship^27,28^. The OMCO collector created three primary types of disturbance: (1) tracks in the seafloor made by the Archimedes screws used for propulsion, (2) removal of sediment and nodules between the screw tracks by the collection equipment and (3) a plume of resuspended sediment released by the action of the collector movement and nodule collection activities. The test created a looping track disturbing an area of roughly 0.4 km^2^ (Fig. 1), which was compared to an undisturbed control area roughly 2 km away (Extended Data Fig. 1).

## Collection tracks

**Fig. 2:**
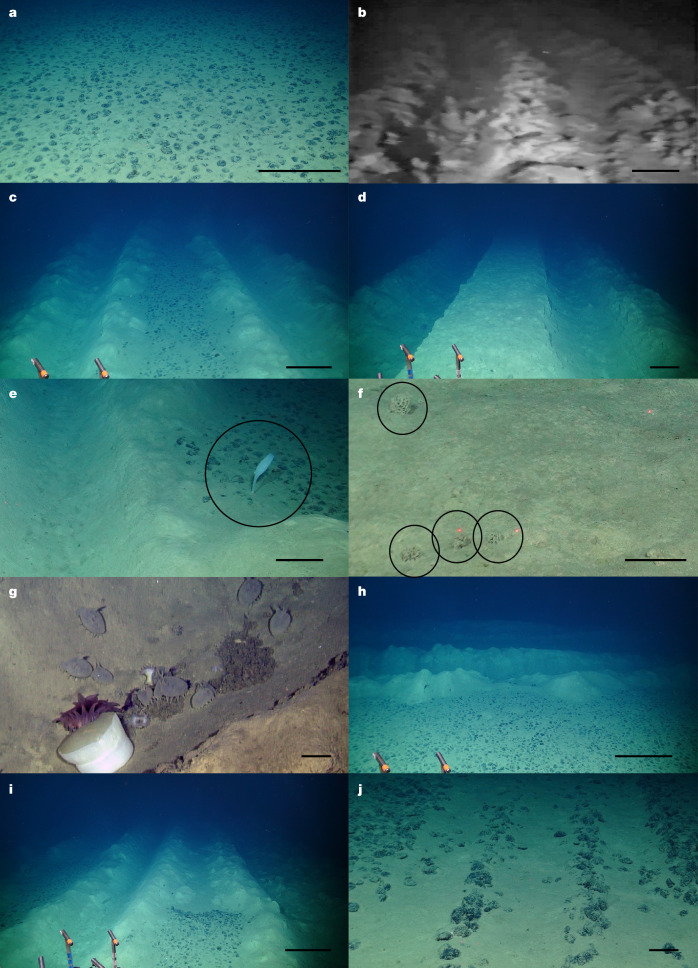
Images of the tracks made by the 1979 OMCO mining test taken in 2023 unless stated otherwise. **a**, Typical photograph of undisturbed seabed at the control site. **b**, Still frame from a video of track taken from the seabed collector in 1979. **c**, Typical view of collector track in 2023, note the low collection efficiency of nodules in the central area. **d**, Area of track with complete removal of nodules in the centre and deeper propulsion tracks either side. **e**, Hexactinellid sponge in the collector track. Sponge in the black circle is roughly 270 mm in height. **f**, Xenophyophores (in the black circle, each 20–50 mm in diameter) growing in the track. **g**, Aggregation of Elpidiid holothurians, anemones and particulate organic matter in the propulsion track. **h**, Area immediately adjacent to the tracks (tracks visible in the background) showing typical nodule densities in area modelled to have been affected by the plume. **i**, Image showing an abrupt change in the nodule collection, probably caused by raising the collector rake. **j**, Epibenthic sled track, probably from 1978, made near the mining collector track. Note scale varies with perspective and each image was taken in a different location so has a different scale. Minor processing of colour balance has been applied to images to correct for differential absorption of light in water. Following image integrity standards, corrections have been applied equally across the entire image and are applied equally to control conditions. Scale bars, 0.5 m (**a**–**d**,**h**,**i**), 0.1 m (**e**–**g**,**j**).

**Fig. 3:**
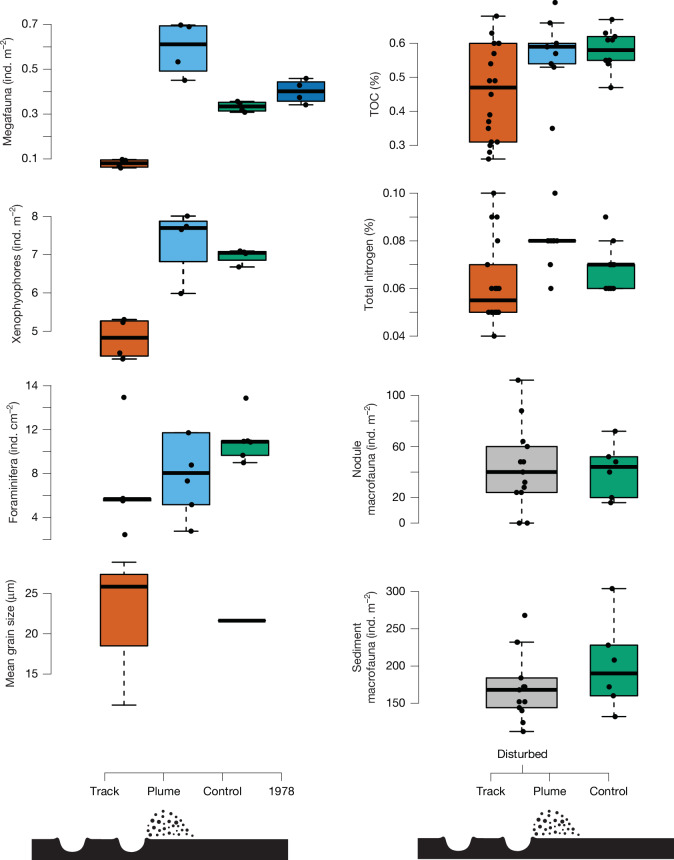
Response of key parameters in 2023 to OMCO disturbance at the track centre (vermillion), plume (roughly 10 m away from track, sky blue), control (roughly 2 km away from track, green) and predisturbance data from 1978 (dark blue). These include megafauna and xenophyophores (both greater than 20 mm) determined from photographic transects (*n* = 4 transects per treatment, covering a total of 5,963 m^2^). Meiofaunal foraminifera from the surface sediments (*n* = 6 cores per treatment). Mean grain size in the surface (top 10 mm) sediments (*n* = 4 cores total). Total organic carbon (TOC) and total nitrogen in the surface (top 5 mm) sediments (*n* = 38 cores total). Sediment and nodule macrofaunal samples (*n* = 6 cores control, *n* = 13 cores disturbed) from the upper 150 mm of the sediment. Note for macrofaunal samples some of the boxcore locations could not be located accurately enough to determine whether they landed on the track or plume area so those in the track area were classified as disturbed (grey). Box plots represent median values, upper and lower quartiles and range with outliers removed. Actual data points are plotted as black circles. Results from the vehicle propulsion tracks were only available for megafauna so were not included on this figure. Note cartoons of disturbance regimes are not to scale. All data used in this figure are available in Supplementary Data 1.

**Extended Data Fig. 2:**
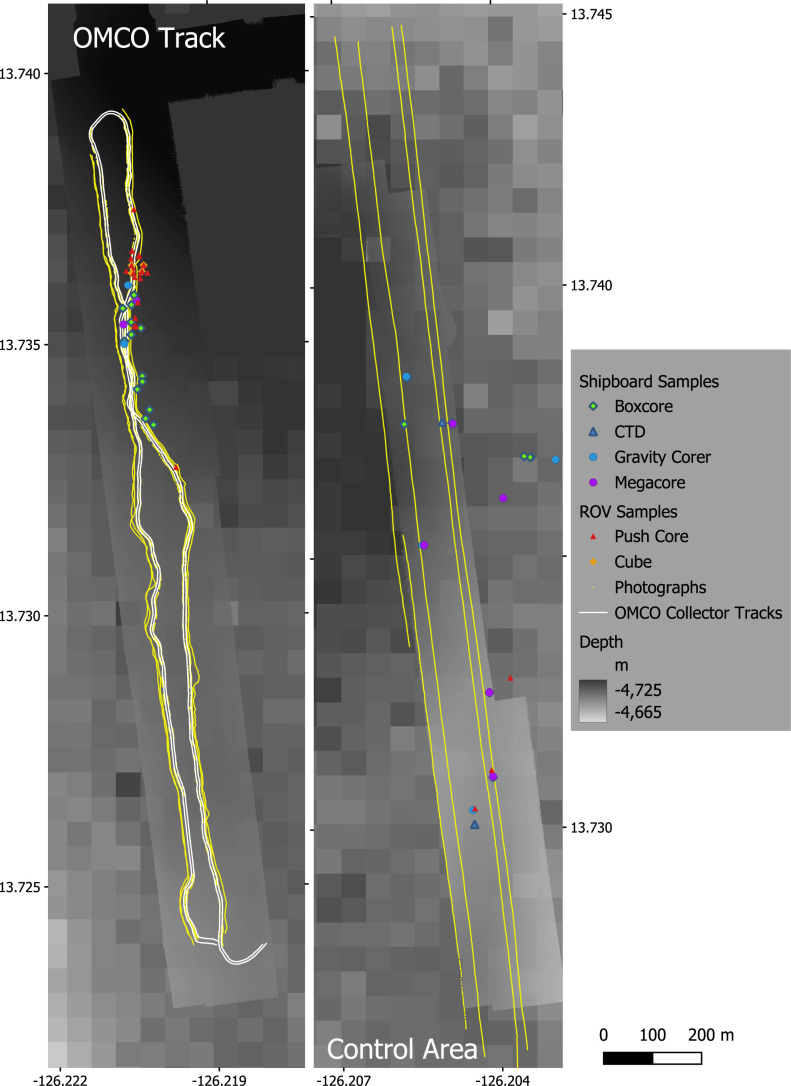
Map of tracks and control area showing the samples obtained for this study. Sample types indicated on the legend. Full details of each sample are in the supplementary data. The yellow points represent the location of ROV imagery transects. Position in decimal degrees (WGS84).

**Extended Data Fig. 3:**
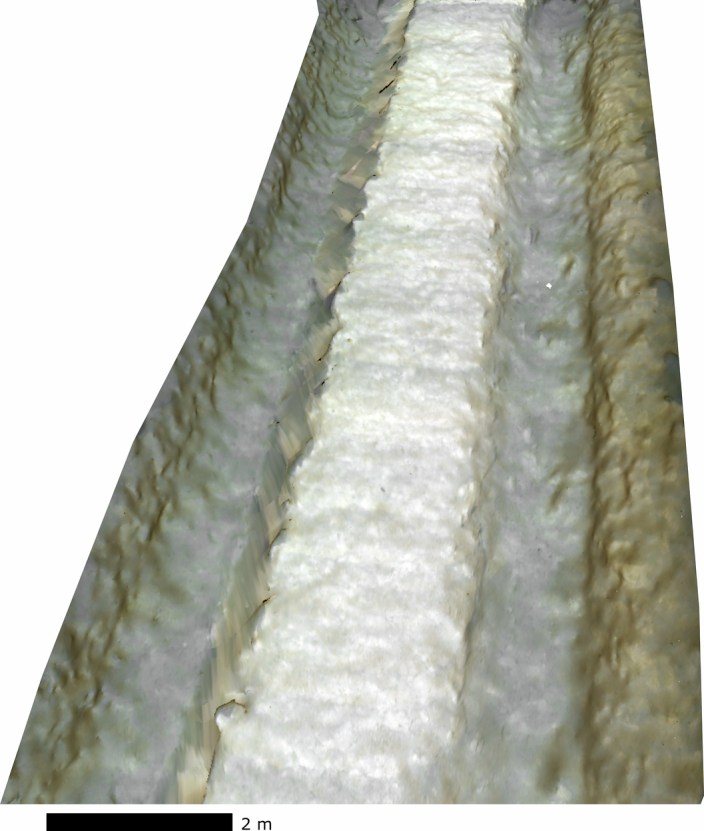
Photomosaic of track. Oblique view of 3-dimensional model.

Between the two propulsion tracks there was a roughly 4.5-m-wide disturbed area that was passed over by the collector rake, which we refer to as the collection track. The physical impact visible in the OMCO collection tracks is similar in appearance to the collection area impacts made by most modern collection vehicles^29^. Our observations (Extended Data Fig. 2) indicate that physical changes to the seafloor have been persistent over 44 years. The collector impacts vary visually, from complete collection of all nodules to no apparent impact on the structure of the nodule-covered seafloor (Fig. 2 and Extended Data Fig. 3). Still visible in 2023, the documented changes were caused during the test and are linked to hydraulic lifting of the collector rake (the likely cause of Fig. 2i) and the depth that the mining machine sank into the seafloor. Some epibenthic sled tracks created in 1978 occur in the same area as the collection tracks and are small in comparison with the collection tracks (Fig. 2j). In the areas where nodules were removed, the sediments within the tracks in 2023 had a similar grain size but generally lower and more variable organic carbon content (Fig. 3) compared with undisturbed areas outside the tracks.

Sediment-dwelling macrofauna, dominated by annelids (43% of total abundance in control), arthropods (34%) and molluscs (18%), were present in slightly lower, but broadly similar, total densities (numbers per unit area) in the disturbed sites in 2023 to the undisturbed control site (Fig. 3, Extended Data Fig. 1 and Extended Data Table 1). Note that the disturbed macrofaunal samples include both the collection track and area immediately adjacent, so our sampling may underestimate the impacts on the track. However, our results correspond with evidence from the more eutrophic DISCOL site, 4,760 km to the south-east of the OMCO site, where sediment-dwelling macrofauna had largely recovered to predisturbance densities in disturbed areas after 7 years (ref. ^25^). Nodule-dwelling macrofauna had similar density in the disturbed area in 2023 compared with the sampled control area, although numbers of nodule dwellers would be expected to reduce if more nodules were removed (only 2 of the 13 disturbed samples had no nodules present and hence no nodule fauna). In a typical mining scenario near complete removal of nodules would probably lead to further reductions in nodule-dwelling faunal density in the tracks.

The collection tracks and propulsion tracks both had abundant xenophyophores 44 years after the test (Fig. 3; primarily an undescribed reticulated spherical species with maximum densities of roughly eight individuals per m^2^) that had colonized even the most visibly disturbed areas of the tracks. These xenophyophores reached sizes of more than 50 mm in diameter and appear to be the first sessile megafaunal-sized organisms recolonizing the mining track disturbance in this area. The role of xenophyophores as early colonists has been predicted^30^ and xenophyophores are able to grow relatively quickly in abyssal sedimentary environments (estimated 1–2 years to reach 50 mm diameter)^31^ but they were not noted in other shorter-term abyssal recovery assessments^22^. The succession dynamics in the mining tracks here may mimic natural disturbance events in the deep ocean associated with extreme sedimentation, for example ash falls^32^, which are also colonized by xenophyophores. Despite the presence of new colonizers in 2023, the overall xenophyophore assemblage was reduced in density in the track areas compared with elsewhere, as several morphotypes common in control areas were present in much lower numbers in the tracks.

In areas of the seafloor visibly disturbed by the collector there were very few sessile megafaunal metazoans present in 2023, despite these being regularly observed in the control area in 2023 and in 1978, before disturbance. Two large (greater than 100 mm) hexactinellid sponges were observed on the collection tracks in 2023, but both were living on nodules that appear to have been undisturbed in 1979 as the collector passed over them. Mobile megafaunal deposit feeders, such as the holothurian *Psychronaetes hanseni* (Pawson, 1983) and the echinoid *Plesiodiadema globulosum* (A. Agassiz, 1898), were observed on the collection tracks in 2023 but overall megafaunal densities were very low on tracks (always less than 0.1 individuals per m^2^) and significantly different from control areas (mean 0.33 individuals per m^2^) and precollection test (mean 0.28 individuals per m^2^; Fig. 3, Supplementary Data 1 and Extended Data Table 1).

Our data indicate that microbial biomass was similar inside and outside the tracks in 2023 (mean 92 mg C m^−2^ (range 32–154) inside versus 52 mg C m^−2^ outside (range 28–77)). Initial estimates suggest total carbon assimilation was considerably higher on the track (mean 0.032 mg C m^−2^ day^−1^ (range 0.026–0.038)) but not statistically different from outside (mean 0.012 mg C m^−2^ day^−1^ (range 0.011–0.014); probably because of the low replication, *n* = 2).

## Vehicle propulsion tracks

**Extended Data Fig. 4:**
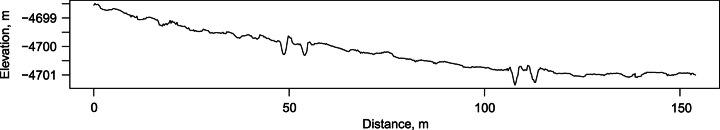
Cross section of multibeam of track area. Units meters. Distance across track shown in meters on x-axis. Depth on y-axis. Figure shows two collector tracks separated by approximately 60 m. Cross section from 800604 E 1519645 N to 800759 E 1519645 N (WGS 84 Universal Transverse Mercator Zone 9 N).

The tracks are very similar in physical appearance between 1979 and 2023 (Fig. 2), which is also observed in other long-term abyssal disturbance experiments^22^ and related to low abyssal sedimentation rates (1.5–11 mm kyr^−1^)^33^. The propulsion created furrows in the seafloor observed to be 0.2–0.8 m deep and 1–3 m wide in 2023 (Extended Data Fig. 4). The action of the propulsion system during the test displaced sediment and created berms on either side of each track, reaching up to 0.5 m in height and extending laterally between 0.5 and 2 m. These berms covered the central area of the track in places and 2023 boxcore samples show that the original surface layer of nodules was still present in places under the raised berm sediments. These propulsion tracks appear to create a considerably larger level of disturbance than planned modern tank-track propelled collection vehicles^17,34^. The OMCO vehicle propulsion tracks appear to trap particulate material in some areas, with visible accumulations of organic detritus and occasional macro plastic items such as plastic bags in 2023. This trapping of material was also observed at DISCOL^35^.

The propulsion tracks harboured a distinct invertebrate megafaunal community in 2023 based on image data, composed of only 35 taxa from three phyla (20 echinoderms, nine cnidarians and six arthropods) and five bony fish morphotypes, compared with undisturbed locations also surveyed in 2023 (total 76 taxa from nine phyla, particularly echinoderms, cnidarians and poriferans). The propulsion track assemblage in 2023 was largely dominated by *P. globulosum* (42% of total abundance; mostly in higher sections at the track edges) and the holothurian Elpidiidae sp. indet. (33%, mostly in central sections at the bottom of the track). At least five taxa were aggregated in the tracks in 2023 (Fig. 2g), particularly Elpidiidae sp. indet. and Actiniaria fam. indet., potentially benefiting from enhanced organic material patchily deposited across the track^35^. A few specimens of the sessile black coral *Schizopathes affinis* sp. inc. longer than 50 mm were observed growing directly on sediment in the middle of the track 44 years after disturbance. Most nodules were removed, probably through displacement or burial, but a few of the remaining nodules were populated in 2023 by two anemone species that were common in non-affected areas. Densities of megafauna in the propulsion tracks in 2023 were intermediate between the collection track and the similar levels at the control and preimpact areas (map in Extended Data Fig. 1). This shift in propulsion track assemblage community structure since disturbance, with low richness and high dominance, is consistent with a classic disturbance and organic enrichment scenario^36^, a local pattern not yet seen in other areas of the CCZ^10^. The potential organic enrichment found in these experimental test tracks is unlikely to be as obvious with the more extensive track formation in a future exploitation scenario. First, collector propulsion at present appears to be favouring tracked design, which will not cause such deep propulsion tracks. Second, the available organic material will be spread between all depressions in the area, meaning that each gets a smaller amount with many disturbances.

## Plume area

**Extended Data Fig. 5:**
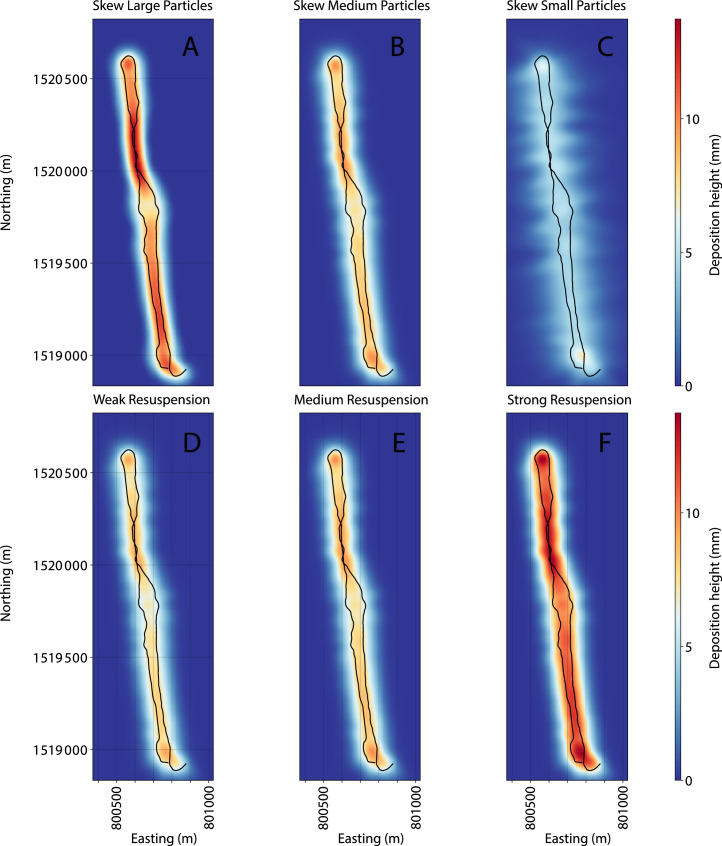
Plume model results and uncertainty plots. A range of particle settling speeds (top) and sediment mobilization (bottom) were considered based on the sediment characteristics measured at the site, to investigate the possible range of deposition patterns. A. Deposition after sediment from upper 7 cm and 10 % furrow area of Archimedes screws resuspended (5.1 kg m^−3^). B. Deposition after sediment from upper 5 cm and 5 % furrow area of Archimedes screws resuspended (2.6 kg m^−3^). C. Deposition after sediment from upper 3 cm and 1 % furrow area of Archimedes screws resuspended (2.31 kg m^−3^). Intermediate deposition scenario with resuspension from settling velocities of D. 0.1 mm s^−1^, E. 1 mm s^−1^ and F. 3.5 mm s^−1^. Coordinates are eastings and northings (meter units) WGS 84 UTM Zone 9 North.

Obvious clouds of sediment were observed in the underwater video obtained from the collector in 1979 but no quantitative measurements were made. The local deposition pattern of the sediment plume caused by the operations of the OMCO collector was estimated from a turbidity current model similar to that validated by observations^17^. For the typical scenario, the model results (Fig. 1) predict deposition thicknesses ranging from 0 to 10 mm over a distance of tens of metres away from the tracks (for the range, see Extended Data Fig. 5). The highest deposition thicknesses occur between the two tracks and at the turn at the northern extent of the track, where there is overlapping deposition coming from neighbouring track segments.

**Extended Data Fig. 6:**
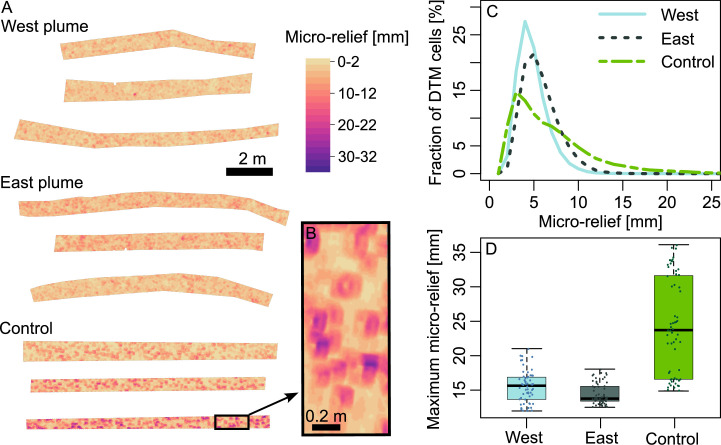
Nodule infill. A. Mapping of micro-relief over 3 photogrammetric transects in locations 5-m west and east to the plume and in the control area. B. Zoom on micro-relief maps showing the presence of elliptic nodules of 5 to 10 cm diameter. C. Distribution of micro-relief values over the digital terrain models (DTM). D. Dispersion of maximum micro-relief per locations. Boxplots represent median values, upper and lower quartiles and range with outliers removed. The (n = 180) individual maximum micro-relief values are plotted as points overlying the boxes. Overall micro-relief in the control area was higher (average 5.9 mm) than the west and east plume areas (average 3.8 and 4.7 mm respectively). Maximum micro-relief was significantly higher in the control (average 24.1 mm) than the east and west plume areas (average 14.4 and 15.3 mm respectively; Kruskal Wallis χ^2^(2) = 89.52, df=2, p < 0.001, n = 180). Wilcoxon post-hoc tests further indicated significant differences between the control and both plume locations (p Bonferonni <e-11, n = 120), but not between plume locations.

In 2023, the area adjacent to the tracks and between pairs of tracks was covered in nodules and was not visually discernible from any other areas outside the track. If we assume that this area would have been covered by resuspended sediments from a plume, then in the 44 years since the test the sediments have either been redistributed in the sediment surrounding the nodules or laterally dispersed, potentially during increased current events^37^ or by bioturbation^33^. Photogrammetric assessment of the 3D nodule surface suggests that there has been significant sediment infill (up to around 10 mm) between the nodules in the area adjacent to the tracks (less than 10 m) compared with the control area (Extended Data Fig. 6). The plume area had similar levels of surface organic material to the control area samples (total organic carbon and total nitrogen in dry sediment; Fig. 3). The plume area had elevated densities of megafauna in 2023, particularly bryozoans (largely Cyclostomatida fam. indet.) and echinoderms (ophiuroids, echinoids and holothurians) compared with both the track and control areas (Fig. 3).

## Background environment

The near-seabed oceanographic conditions are similar between the OMCO test and control areas, with typical CCZ temperature (1.48 °C in situ), oxygen (151 µmol kg^−1^) and absolute salinity (34.87 g kg^−1^) and current speeds twice exceeding the typical tidal range for this area of the CCZ (the major axes of semi- and diurnal M2, K1 components are 13.8, 7.4 mm s^−1^; mean velocity from three lowered Acoustic Doppler Current Profiler (ADCP) profiles 8 m above the seabed: 40 ± 13 mm s^−1^, direction 170 ± 81° was parallel the isobath and roughly aligned with the track orientation). No comparable data are available from 1979. Nodules in the collector area were abundant (from 14 boxcore samples, mean 155 nodules per m^2^ and 18.6 kg nodules per m^2^, counts of 542 nodules) and relatively large (largest dimension, mean 72 ± 19 mm s.d.; maximum observed 152 mm) ranges comparable to other exploration areas within the CCZ^38^. Most nodules were found at the sediment surface. There are no obvious differences in nodule appearance between the 1978 photos and the observations in 2023. Natural sedimentation rates across the CCZ are low (1.5–11.5 mm kyr^−1^)^33^.

## Conclusions

This study is one of the few studies on the impacts caused by an abyssal polymetallic-nodule-mining collector vehicle, and represents a long duration study to determine the extent of recovery from mining disturbance in the CCZ. The areal extent of disturbance in the OMCO test was small relative to a commercial scale mine, with limited distance travelled by the collector and areas with incomplete nodule collection. Compared with current designs of nodule-harvesting vehicles, the OMCO vehicle had a similar mechanical collection approach to some but distinctly different impacts in the propulsion tracks, with Archimedes screws penetrating far more deeply into the seafloor than modern tracked vehicle designs. We show that visible physical disturbance remains in the abyss 44 years after this test with very little visible sign of physical remediation. However, we demonstrate that mobile organisms, including megafauna and macrofauna, are living in the most disturbed areas. As far as we can measure them, sediment and nodule macrofauna densities and microbial biomass are similar in and out of disturbed areas. We also provide evidence of early stages of re-establishment of some sessile megafaunal-sized species after four decades, although megafaunal communities are still very different from past or undisturbed conditions. The depressions in the seabed created by the propulsion tracks appear to have aggregated organic matter and attract mobile megafaunal deposit feeders. Sediments from the plume created by the OMCO collector are no longer obvious but are detectable in photogrammetric measurements of sediment infill between nodules and appear to support elevated densities of megafauna compared with undisturbed areas. Upscaling the observations to the spatial scales and impacts of a full commercial mine will require further work and would also require a better understanding of the natural variability of the CCZ^10^. However, our results show that, if mining were to take place, efforts to reduce the direct collector impact could be effective in limiting the overall ecological effects of mining operations on seafloor biota, but the visible physical effects of the collection can be assumed to last for at least many decades.

## Methods

### Mining test

Between 15 and 18 March 1979 an experimental mining machine was deployed on a site centred 13° 44′ N 126° 13.5′ W. The 9-m wide, 14-m long, 4.5-m high machine^40^ was lowered to the seafloor at around 4,700 m deep from the moon pool of the *Hughes Glomar Explorer* vessel by extending a steel riser, made up of 60-foot (18-m) pipe sections^27^. This riser was attached to a 150-ton buffer^41^, which was then connected to the vehicle by a flexible linkage (umbilical) with electrical cable, hydraulic lines and a nodule slurry hose. On the seafloor, the buffer was positioned roughly 20 m above and ahead of the mining vehicle. When it landed on the seafloor, the collector vehicle was used to mine an unknown quantity of nodules over the 4 days of the experiment. The collector was self-propelled, using two Archimedes screws of around 2 m diameter, which achieved speeds over the seabed of 0.25–1 m s^−1^ (ref. ^42^). The vehicle was driven around 1 nautical mile (1.85 km) in a roughly northerly direction. It did a 180° looped turn and proceeded southwards, doing some manoeuvrability tests. The vehicle collected nodules mechanically using a rotating seabed rake that picked up nodules and transferred them by using a conveyor to a crusher. The crushed nodule slurry was pumped through the flexible linkage to temporary storage in the buffer. The nodule slurry could then be lifted through the riser pipe string to the surface vessel using an airlift, electric pump and pressurized water system^28^. On at least one occasion, the nodules formed a blockage and the rake was lifted stopping nodule collection. Previous nodule collection had been carried out with epibenthic sleds at the site. The vehicle position was recorded with reference to a long baseline acoustic array, informed by eight transponder beacons around the site. The array provided good relative navigation: absolute navigation was provided by an early satellite navigation system. The location of the test estimated at the time was accurate to within roughly 500 m of the known modern position.

### Predisturbance photography

Photographs were obtained of the seafloor in the area of the collector test during three cruises of the RV *Governor Ray*^43^. Two cruises were carried out before the test (June 1978, GR7801; November 1978, GR7804) and one after (October 1979, GR7904). Monochrome images were collected using a Benthos 35 mm film camera, mounted vertically on a towed frame. Height above the seabed was determined with a Benthos Model 211 altimeter and recorded on each photographic frame. Images in which seafloor was visible were collected at altitudes ranging from 0.6 to 9 m. Only images collected at altitudes less than 3.5 m were included in the analysis as this allowed reliable detection of megafaunal specimens larger than 20 mm. Overlapping images were removed through manual inspection (leaving a total of 1,929 images that could be analysed, available from ref. ^44^). Although the tracks were not imaged in this survey, the photographs provide important context about change in the baseline environment over time and are used to assess megafaunal communities.

### Sample collection

Samples, imagery and other data were collected during RRS *James Cook* expedition JC241 between 14 February and 12 March 2023 (see Supplementary Data 1 for metadata, data summary and full data). The expedition was centred on the area of the 1979 collector test. This area is not within any of the current International Seabed Authority exploration contract areas but is located 1–3 km south of the central of the three areas now contracted to the Cook Islands Investment Corporation. Four treatment classes were chosen for assessment in 2023, each expected to have a different type of disturbance during the OMCO test: (1) collection tracks, 4.5-m wide areas effectively mined by the collector rake with evident mechanical disturbance, flat surface and nodule depleted; (2) vehicle propulsion tracks, 0.2–0.8-m deep and up to 2-m-wide parallel furrows each side of the collection tracks with severe mechanical disturbance still evident, concave-shaped and very few nodules visible; (3) plume areas, adjacent (5–10 m) to vehicle tracks, with no apparent disturbance, mostly flat and harbouring high nodule abundance and (4) the control site, which assumed the non-affected area located roughly 2 km east from the OMCO test area but with similar terrain features, particularly being mostly flat and harbouring high nodule abundance. The deep propulsion tracks could not be physically sampled as the remotely operated vehicle (ROV) had insufficient reach to core them or pick up faunal specimens.

Multibeam sonar data (Reson 7125), photographic (Insite Super Scorpio and National Oceanography Centre (NOC) AESA Camera) and video (Insite Mini Zeus Mk2, Kongsberg Eyeball Cam) imagery and samples of seafloor sediments, nodules and animals were acquired using the UK ROV *ISIS*. ROV relative navigation used RDI Navigator 300 kHz bottom tracking Doppler velocity log (DVL). Lowered gear was equipped with a Sonardyne Ranger2 Ultra-Short Baseline (USBL) acoustic beacon to ensure accurate positioning. ROV multibeam echosounder data were recorded in PDS2000 and processed using Qimera v.2.4.3. USBL positions were updated with DVL navigation and tidal corrections were applied. Multibeam echosounder backscatter processing was carried out in FMGT v.7.10.1, based on the processed bathymetry files. Further navigational adjustments to compensate for DVL drift were applied in ArcMap v.10.6, where final merged grids were created.

Sediment samples were obtained with a 50 × 50 cm USNEL Boxcore (*n* = 19)^45^, Bowers and Connelly Megacore (*n* = 8; 100-mm-diameter tubes), ROV push cores (*n* > 38; 55 mm diameter) and a 5-m gravity core (*n* = 6; 70 mm diameter) targeted at random within specific features. The position of some boxcores, megacores and gravity cores relative to the tracks was determined from ROV images of their imprints but not all could be located this way. Megafauna were sampled by direct collection with the ROV. Fishes were sampled using a fish trap lander^46^.

Boxcores provided quantitative samples of nodule and sediment macrofauna. The boxcore was equipped with a USBL positioning beacon and every effort was made to precisely target specific features. The position of some boxcores relative to the tracks was determined from ROV images but not all could be located this way. Because there was some uncertainty in positioning (estimated ±5 m), we could not accurately determine whether each boxcore had landed within the collector track. As a result, we grouped together all samples that were obtained within 10 m from the track as being ‘disturbed’ and compared them to control conditions. This was a different approach from the other analyses, where the samples could be accurately positioned relative to the track with the ROV imagery and precision collections.

To process the boxcores, nodules were removed and nodule fauna immediately sorted and preserved. Sediment fauna were extracted from the upper 100 mm of sediment from the entire core and overlying water by sieving through a 300-µm sieve with cold filtered seawater. A 15 × 15-cm subcore was taken and sliced in two layers, 0–20 and 20–50 mm, before being sieved through a 300-µm sieve, live sorted, photographed and preserved individually in 80% non-denatured ethanol. All remaining sediment in the boxcore was sliced in 0–20, 20–50 and 50–100-mm layers and sieved on 300-µm sieves, before being bulk fixed in 100% non-denatured ethanol^47^. For the quantitative analysis, all data from the 15 × 15-cm subcore were combined with the main sample. Individuals were identified to phylum level for this analysis. Arthropods and annelids were only counted if the head was present, and echinoderms only if the oral disc was present. For the macrofaunal densities, the numbers of organisms in the whole boxcore were presented (adding the live sort numbers to those counted from the rest of the core). Any nodule-dwelling fauna recovered in any of the sediment layers were excluded from both the sediment and nodule fauna analyses to avoid inconsistency. For the sediment macrofauna, any pelagic (for example, chaetognaths, appendicularians, ctenophores) or meiofauna (for example, halicarids, ostracods and copepods) were excluded from the analyses.

Meiofaunal foraminifera were sampled using ROV push cores to enable precise sampling in the collection track, plume area and the control site. For meiofaunal foraminifera assessment, cores were extruded on board at 10-mm intervals and preserved in 70% non-denatured ethanol. Samples were sieved through a 63-µm mesh, and foraminifera from the top 10 mm were identified, counted, photographed and differentiated as live or dead using a Leica M205c microscope with a 32MP sensor camera.

Total bacterial biomass (mg C m^−2^) and assimilation (mg ^13^C m^−2^) of ^13^C were assessed through stable-isotope pulse chase experiments in 2023. Four benthic incubation chambers were deployed: two on the track and two roughly 20 m away on sediment with no visible disturbance. The off-track samples were within the area expected to have been affected by the plume. A total of 0.2 g of isotope-labelled *Phaeodactylum tricornutum* (grown in media with 25% ^13^C and ^15^N) was injected into each chamber. After roughly 70 h, the incubation chambers were removed and ROV push cores were used to sample the sediments in the imprint (*n* = 3 per chamber). The upper 20 mm of sediment were sampled and frozen. The total bacterial biomass and assimilation were calculated from the concentration of and label incorporation into the bacterial fatty acids (IC_15:0_) following standard methods for extraction and processing^48^. These used an average fraction-specific bacterial to phospholipid fatty acid (PLFA) ratio encountered in sediment dominated by bacteria (0.017). The ^13^C assimilation values (mg ^13^C m^−2^) for bacteria were then converted to daily C assimilation rates (mg C m^−2^ day^−1^) by accounting for the fractional abundance of ^13^C in the added algae (3.9 atom%) as follows: (C assimilation = ^13^C incorporated (mg ^13^C m^−2^)/fractional abundance of ^13^C in algae)/2.8.

Assessment of total nitrogen, total carbon and total organic carbon was done from the top 5 mm of sediment megacores and ROV push cores. These cores were sliced, stored in foil lined Petri dishes and frozen (−20 °C). In the laboratory sediments were lyophilized and homogenized before analyses. Samples were analysed following acid vapour treatment (HCl; 12 h), using a FlashSmart elemental analyser (Thermo Scientific). A four-point daily calibration was performed using differing weights of High Organic Sediment Standard OAS and Low Organic Soil Standard OAS (Elemental Microanalysis Ltd, NIST certified values). The standards were then analysed as unknowns during the beginning, middle and end of the run to check for precision. The results of the unknowns were within the uncertainty limits of the certified values that are high organic standard (carbon 7.17 ± 0.09%, nitrogen 0.57 ± 0.02%), low organic standard (carbon 1.65 ± 0.02%, nitrogen 0.14 ± 0.01%), with detection limits of 100 ppm for both C and N.

Sediment grain size was measured by laser diffraction (Malvern Mastersizer) following the same approach as ref. ^49^.

Measurements and samples of the water column were made using a CTD Rosette with 24 10-l Niskin Bottles, the Seabird SBE 9plus CTD Unit and RDI 300 kHz lowered ADCP.

### Plume modelling

A turbidity current box model with settling^50^ was used to calculate plume deposition on the basis of the operational parameters of the OMCO test and the results of studies into gravity currents from moving sources^51^. A range of particle settling speeds and sediment mobilization were considered based on the sediment characteristics measured at the site, to investigate the possible range of deposition patterns. Three amounts of suspended sediment across the range of scenarios were considered. These corresponded to the upper 3, 5 and 7 cm of the seabed being resuspended by the rake along with 1, 5 and 10% of the furrow area created by the Archimedes screws, to cover a range of potential scenarios. This equated to discharge concentrations of 2.31, 2.6 and 5.1 kg m^−^^3^. In addition, three distributions of settling velocity were considered, with the slowest scenario dominated by velocities around 0.1 mm s^−1^ and the fastest scenario dominated by velocities around 3.5 mm s^−1^. The results presented (Fig. 1) correspond to an intermediate scenario across all parameters. The model deposition thickness did not exceed 15 mm or extend more than 100 m from the tracks in any scenarios investigated (Extended Data Fig. 5) and so the intermediate scenario was considered a reasonable representation.

### Photography and track assessment in 2023

Scalable, high-resolution image and video transects of the seafloor were obtained using several camera systems mounted on the ROV *ISIS* across the OMCO test and control areas (Extended Data Figs. 1–3). Vertically facing photographs were collected using a Grasshopper2 GS2-GE-50S5C camera in the collection tracks, plume area and control site^52^ while high-definition oblique video transects were collected using a Super-SCORPIO HDR-CX560V camera to characterize the community within vehicle propulsion tracks^53^. All image and video data were collected at a target altitude of 2.5 m above the seabed.

### Image processing

Overlapping seabed areas surveyed during the two ROV imaging surveys were removed from analyses, based on USBL and DVL navigation data, so faunal counts were not repeated. Overlapping areas of still and video images were checked before removal. For the video analysis the area surveyed was a fixed width of 2 m, and only sections where the full width of the propulsion track was visible were retained for subsequent numerical analyses. Only still images from the collection tracks where nodules had been cleared were used to calculate ‘Track’ densities. Megafauna specimens larger than 20 mm could be consistently detected and were counted in both still image sets and videos. These were counted, measured and identified using BIIGLE v.2.0 software^54^ from both the precollection and 2023 images in the same way by the same annotator. Animals were identified to the lowest taxonomic level possible (morphotype, typically genus or family level in undescribed species) in accordance with the code-based Abyssal Pacific Standardized Megafauna Atlas v.1. (APSMA, available in ref. ^55^). The APSMA catalogue follows open nomenclature^56^ to report the taxonomic resolution reached in each taxon but all morphotypes identified from the catalogue were deemed as sufficiently different by taxonomic experts to be confidently considered separate species. Taxa living in a closed shell or tube (for example, most polychaetes) were excluded from analysis as it is not possible to determine whether these are alive from imagery data. Image annotations were quality controlled by random checks of previously annotated images and several checks for consistency of identifications were made using the Label Review Grid Overview tool. Sample units were composed of images randomly selected without replacement until a consistent seabed area was reached (1,200 m^2^, equating to roughly 1,060 images).

### Photogrammetry

To evaluate whether plume deposition could reduce nodule elevation, micro-relief differences were tested in the control, and 5 m to the east and west of the tracks. For each location, three random sets of 100 consecutive images were selected to produce replicates of 10-m seabed portions with 3D photogrammetric models of the seabed (millimetre resolution, Agisoft Metashape, v.2.0.1). Models were scaled with navigation, altitude and attitude of the ROV. For each model, a centimetre-resolution georeferenced digital terrain model (DTM) was imported in R as raster. Local standard deviation of bathymetry (that is, micro-relief) was calculated with a moving window of 9 × 9 cm^2^ (that is, roughly the area of a single nodule) using the function adjSD (package MultiscaleDTM, v.0.8.3, ref. ^57^; Extended Data Fig. 6a,b). For each transect, DTM micro-relief was randomly binned into 20 subsamples per transect without replacement. Maximum micro-relief value was retrieved from each subsample (*n* = 180) to isolate the larger height difference between seabed sediment and nodules. Maximum micro-relief differences among locations were tested with non-parametric Kruskal–Wallis and post hoc Wilcoxon tests. Micro-relief may be associated with plume material discharged by the OMCO collector (shown as ref. ^39^ in Fig. 1).

### Inclusion and ethics statement

All collaborators of this study fulfilling the criteria for authorship required by Nature Portfolio journals have been included as authors, as their participation was essential for the design and implementation of the study. This work was carried out in the Area, beyond national jurisdiction, and has no clear local partners. Local and regional research relevant to our study was taken into account in citations.

### Reporting summary

Further information on research design is available in the Nature Portfolio Reporting Summary linked to this article.

## Online content

Any methods, additional references, Nature Portfolio reporting summaries, source data, extended data, supplementary information, acknowledgements, peer review information; details of author contributions and competing interests; and statements of data and code availability are available at 10.1038/s41586-025-08921-3.

## Supplementary information

Supplementary Data 1 Excel file with data used in paper. Includes the metadata table (Metadata) for all samples collected in OMC track site on expedition JC241. A summary (Summary Data) is included, which includes the variables, units, sample types and group averages, numbers of samples and standard deviations. Worksheets containing detailed datasets on sediment chemistry (TOC_TN), grain size (Grain Size), sediment macrofauna (SedimentMacrofauna), nodule macrofauna (NoduleMacrofauna), microbes (Microbes), meiofaunal foraminifera (MeiofaunalForaminifera) and megafauna (Megafauna) are provided. These data are used in the figures and other assessments in this study.

Reporting Summary

## Figures and Tables

**Extended Data Table 1:**
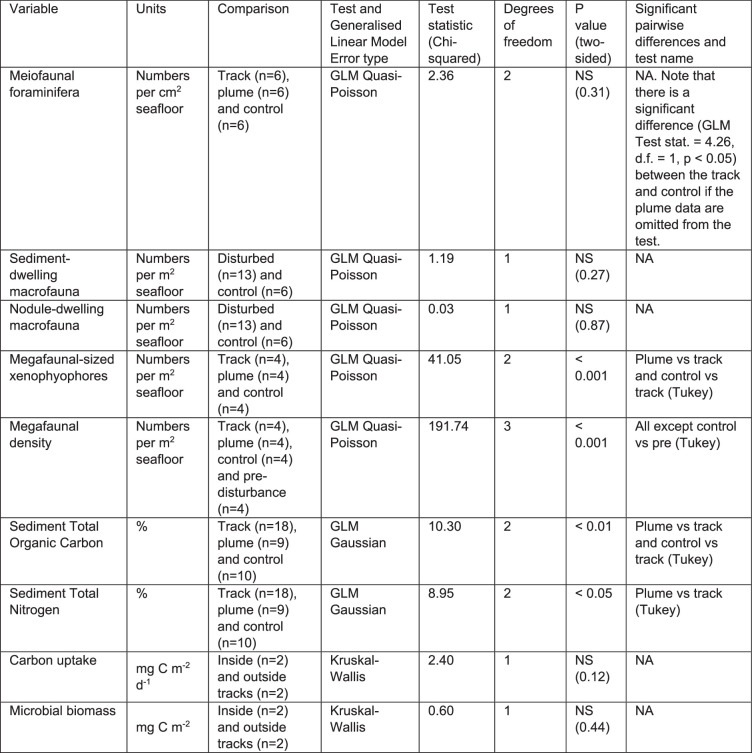
Results of statistical tests for environmental parameters quantified in OMCO collector tracks, plume area and control site

NS = not significant (p > 0.05). NA = Not applicable. Generalized linear (GLM) statistical models were independently developed using R (version 4.4.1) to examine whether the variable under investigation (e.g. faunal density) could be explained using the explanatory variable of disturbance type (categorical, e.g. track, plume and control). Quasi-Poisson errors were used for count variables, where variance appeared to be related to the mean. Gaussian errors were used for continuous data and selected after verifying normality (by visual examination of quantile-quantile plot) and homogeneity of variance (by visual examination of conditional boxplots). Kruskal-Wallis was used where variables were not clearly normally distributed. Numbers of observations (n) are indicated in the comparisons column. Note very low replication (n = 2) in microbial carbon uptake and biomass, where tests should be treated with caution. All measurements were made from distinct samples.

## Data Availability

Data generated for this study are available in the Supplementary Information. The images of the seafloor used for analysis are available at 10.5285/2392b266-126b-db3f-e063-7086abc0fe00 (ref. ^52^, images taken in 2023) and 10.5285/27e550f2-81ff-6bf8-e063-7086abc04f4f (ref. ^44^, images taken in the 1970s). Highlight images from the expedition, including many views of the seafloor, tracks and fauna are provided at 10.5285/2e5a5010-4abd-2beb-e063-7086abc0b159 (ref. ^58^). The APSMA image-based taxonomical catalogue used in the identification of organisms in this study is available at Zenodo (10.5281/zenodo.7765164)^55^. Data handling and analyses were implemented using standard methods, software tools and functions detailed in the Methods.

## References

[1] Pickens, C., Lily, H., Harrould-Kolieb, E., Blanchard, C. & Chakraborty, A. From what-if to what-now: status of the deep-sea mining regulations and underlying drivers for outstanding issues. *Mar. Policy*10.1016/j.marpol.2023.105967 (2024).

[2] Jones, D. O. B., Amon, D. J. & Chapman, A. S. A. In *Natural Capital and Exploitation of the Deep Ocean* (eds Baker M. et al.) Ch. 5, 91–110 (Oxford Univ. Press, 2020).

[3] Jones, D. O. B. et al. Biological responses to disturbance from simulated deep-sea polymetallic nodule mining. *PLoS ONE* **12**, e0171750 (2017). 28178346 10.1371/journal.pone.0171750 PMC5298332

[4] Vanreusel, A., Hilario, A., Ribeiro, P. A., Menot, L. & Arbizu, P. M. Threatened by mining, polymetallic nodules are required to preserve abyssal epifauna. *Sci. Rep.* **6**, 26808 (2016). 27245847 10.1038/srep26808 PMC4887785

[5] Van Dover, C. L. et al. Biodiversity loss from deep-sea mining. *Nat. Geosci.* **10**, 464–465 (2017).

[6] Kaikkonen, L. & van Putten, I. We may not know much about the deep sea, but do we care about mining it? *People Nat.* **3**, 843–860 (2021).

[7] Hein, J. R., Koschinsky, A. & Kuhn, T. Deep-ocean polymetallic nodules as a resource for critical materials. *Nat. Rev. Earth Environ.* **1**, 158–169 (2020).

[8] Stewart, E. C. D. et al. Biodiversity, biogeography, and connectivity of polychaetes in the world’s largest marine minerals exploration frontier. *Divers. Distrib.* **29**, 727–747 (2023).

[9] Gooday, A. J. et al. Five new species and two new genera of xenophyophores (Foraminifera: Rhizaria) from part of the abyssal equatorial Pacific licensed for polymetallic nodule exploration. *Zool. J. Linn. Soc.* **183**, 723–774 (2017).

[10] Simon-Lledó, E. et al. Carbonate compensation depth drives abyssal biogeography in the northeast Pacific. *Nat. Ecol. Evol.* **7**, 1388–1397 (2023). 37488225 10.1038/s41559-023-02122-9 PMC10482686

[11] Snelgrove, P. V. R. & Smith, C. R. A riot of species in an environmental calm: the paradox of the species-rich deep-sea floor. *Oceanogr. Mar. Biol. Annu. Rev.* **40**, 311–342 (2002).

[12] Rabone, M. et al. How many metazoan species live in the world’s largest mineral exploration region? *Curr. Biol.* **33**, 2383–2396.e5 (2023). 37236182 10.1016/j.cub.2023.04.052

[13] Danovaro, R. et al. Ecological variables for developing a global deep-ocean monitoring and conservation strategy. *Nat. Ecol. Evol.* **4**, 181–192 (2020). 32015428 10.1038/s41559-019-1091-z

[14] Sharma, R. In *Deep-Sea Mining: Resource Potential, Technical and Environmental Considerations* (ed. Sharma, R.) 229–256 (Springer, 2017).

[15] Madureira, P., Brekke, H., Cherkashov, G. & Rovere, M. Exploration of polymetallic nodules in the area: reporting practices, data management and transparency. *Mar. Policy* **70**, 101–107 (2016).

[16] Stenvers, V. I. et al. Experimental mining plumes and ocean warming trigger stress in a deep pelagic jellyfish. *Nat. Commun.* **14**, 7352 (2023). 37990021 10.1038/s41467-023-43023-6 PMC10663454

[17] Muñoz-Royo, C., Ouillon, R., El Mousadik, S., Alford, M. H. & Peacock, T. An in situ study of abyssal turbidity-current sediment plumes generated by a deep seabed polymetallic nodule mining preprototype collector vehicle. *Sci. Adv.* **8**, eabn1219 (2022). 36129971 10.1126/sciadv.abn1219 PMC9491711

[18] Muñoz-Royo, C. et al. Extent of impact of deep-sea nodule mining midwater plumes is influenced by sediment loading, turbulence and thresholds. *Commun. Earth Environ.* **2**, 148 (2021).

[19] Weaver, P. P. E. et al. Assessing plume impacts caused by polymetallic nodule mining vehicles. *Mar. Policy* **139**, 105011 (2022).

[20] Vos, M. et al. The Asymmetric Response Concept explains ecological consequences of multiple stressor exposure and release. *Sci. Total Environ.* **872**, 162196 (2023). 36781140 10.1016/j.scitotenv.2023.162196

[21] Lutz, M. J., Caldeira, K., Dunbar, R. B. & Behrenfeld, M. J. Seasonal rhythms of net primary production and particulate organic carbon flux to depth describe the efficiency of biological pump in the global ocean. *J. Geophys. Res.* **112**, C10011 (2007).

[22] Simon-Lledó, E. et al. Biological effects 26 years after simulated deep-sea mining. *Sci. Rep.* **9**, 8040 (2019). 31142831 10.1038/s41598-019-44492-w PMC6541615

[23] Stratmann, T. et al. Abyssal plain faunal carbon flows remain depressed 26 years after a simulated deep-sea mining disturbance. *Biogeosciences* **15**, 4131–4145 (2018).

[24] de Jonge, D. S. W. et al. Abyssal food-web model indicates faunal carbon flow recovery and impaired microbial loop 26 years after a sediment disturbance experiment. *Prog. Oceanogr.* **189**, 102446 (2020).

[25] Borowski, C. Physically disturbed deep-sea macrofauna in the Peru Basin, southeast Pacific, revisited 7 years after the experimental impact. *Deep-Sea Res. II* **48**, 3809–3839 (2001).

[26] Levin, L. A., Amon, D. J. & Lily, H. Challenges to the sustainability of deep-seabed mining. *Nat. Sustain.* **3**, 784–794 (2020).

[27] Welling, C. G. An advanced design deep sea mining system. In *Proc. Offshore Technology Conference*10.4043/4094-MS (1981).

[28] Welling, C. G. et al. Ocean mining system and process. US patent 4232903 A (1980).

[29] Kang, Y. & Liu, S. The development history and latest progress of deep-sea polymetallic nodule mining technology. *Minerals* **11**, 1132 (2021).

[30] Gooday, A. J., Durden, J. M. & Smith, C. R. Giant, highly diverse protists in the abyssal Pacific: vulnerability to impacts from seabed mining and potential for recovery. *Commun. Integr. Biol.* **13**, 189–197 (2020). 33312334 10.1080/19420889.2020.1843818 PMC7714518

[31] Gooday, A. J., Bett, B. J. & Pratt, D. N. Direct observation of episodic growth in an abyssal xenophyophore (Protista). *Deep-Sea Res. I Oceanogr. Res. Pap.* **40**, 2131–2143 (1993).

[32] Hess, S. et al. Monitoring the recolonization of the Mt Pinatubo 1991 ash layer by benthic foraminifera. *Mar. Micropaleontol.* **43**, 119–142 (2001).

[33] Volz, J. B. et al. Natural spatial variability of depositional conditions, biogeochemical processes and element fluxes in sediments of the eastern Clarion–Clipperton Zone, Pacific Ocean. *Deep-Sea Res. I* **140**, 159–172 (2018).

[34] Zhang, X. et al. A review on underwater collection and transportation equipment of polymetallic nodules in deep-sea mining. *J. Marine Sci. Eng.* **12**, 788 (2024).

[35] Hoving, H.-J. et al. Major fine-scale spatial heterogeneity in accumulation of gelatinous carbon fluxes on the deep seabed. *Front. Marine Sci.*10.3389/fmars.2023.1192242 (2023).

[36] Pearson, T. H. & Rosenberg, R. Macrobenthic succession in relation to organic enrichment and pollution of the marine environment. *Oceanogr. Mar. Biol. Annu. Rev.* **16**, 229–311 (1978).

[37] Aleynik, D., Inall, M. E., Dale, A. & Vink, A. Impact of remotely generated eddies on plume dispersion at abyssal mining sites in the Pacific. *Sci. Rep.* **7**, 16959 (2017). 29208985 10.1038/s41598-017-16912-2 PMC5717004

[38] Jones, D. O. B. et al. Environment, ecology, and potential effectiveness of an area protected from deep-sea mining (Clarion Clipperton Zone, abyssal Pacific). *Prog. Oceanogr.* **197**, 102653 (2021).

[39] Cheng, Y., Dai, Y., Zhang, Y., Yang, C. & Liu, C. Status and prospects of the development of deep-sea polymetallic nodule-collecting technology. *Sustainability* **15**, 4572 (2023).

[40] Cromie, W. J. Vacuuming the ocean floor. *Technology Illustrated* 33–39 (August/September, 1982).

[41] Brink, A. N. & Chung, J. S. Automatic position control of A30,000 tons ship during ocean mining operations. In *Proc. Offshore Technology Conference*10.4043/4091-MS (1981).

[42] Chung, J. S. Manganese nodule miners on 18,000-ft deep seabed: touchdown, track-keeping control and disturbed seabed track history. *Int. J. Offshore Polar Eng.* **31**, 385–394 (2021).

[43] Morgan, C. L., Nichols, J. A., Selk, B. W., Toth, J. R. & Wallin, C. Preliminary analysis of exploration data from Pacific deposits of manganese nodules. *Mar. Georesour. Geotechnol.* **11**, 1–25 (1993).

[44] Benthic Images Recorded by a Towed Camera in 1978-79 in the Ocean Minerals Company (OMCO) area of the Clarion Clipperton Zone (Pacific Ocean). Published Data Library (National Oceanography Centre, British Oceanographic Data Centre, 2025); 10.5285/27e550f2-81ff-6bf8-e063-7086abc04f4f.

[45] Hessler, R. R. & Jumars, P. A. Abyssal community analysis from replicate box cores in the central North Pacific. *Deep Sea Res. Oceanogr. Abstr.* **21**, 185–209 (1974).

[46] Hartl, M. G. J., Baumann, L. M. & Sweetman, A. K. At-sea application of the comet assay to a deep-sea fish. *Deep-Sea Res. I* **208**, 104298 (2024).

[47] Glover, A., Dahlgren, T., Wiklund, H., Mohrbeck, I. & Smith, C. An end-to-end DNA taxonomy methodology for benthic biodiversity survey in the Clarion–Clipperton Zone, Central Pacific Abyss. *J. Marine Sci. Eng.* **4**, 2 (2016).

[48] Sweetman, A. K. et al. Impacts of exotic mangrove forests and mangrove deforestation on carbon remineralization and ecosystem functioning in marine sediments. *Biogeosciences* **7**, 2129–2145 (2010).

[49] Simon-Lledó, E. et al. Megafaunal variation in the abyssal landscape of the Clarion Clipperton Zone. *Prog. Oceanogr.* **170**, 119–133 (2019). 30662100 10.1016/j.pocean.2018.11.003 PMC6325340

[50] Harris, T. C., Hogg, A. J. & Huppert, H. E. Polydisperse particle-driven gravity currents. *J. Fluid Mech.* **472**, 333–371 (2002).

[51] Ouillon, R., Kakoutas, C., Meiburg, E. & Peacock, T. Gravity currents from moving sources. *J. Fluid Mech.* **924**, A43 (2021).

[52] Benthic Images Recorded by a Remotely Operated Vehicle Stills Camera During Cruise JC241 in the Clarion–Clipperton Zone (Pacific Ocean, 2023). Published Data Library (National Oceanography Centre, British Oceanographic Data Centre, 2025); 10.5285/2392b266-126b-db3f-e063-7086abc0fe00.

[53] Benthic Images Recorded by a Remotely Operated Vehicle Video Camera During Cruise JC241 in the Eastern Clarion–Clipperton Zone (Pacific Ocean, 2023). Published Data Library (National Oceanography Centre, British Oceanographic Data Centre, 2025); 10.5285/2de087c9-cee3-87f4-e063-7086abc0f9a9.

[54] Langenkämper, D., Zurowietz, M., Schoening, T. & Nattkemper, T. W. BIIGLE 2.0—browsing and annotating large marine image collections. *Front. Marine Sci.*10.3389/fmars.2017.00083 (2017).

[55] Simon-Lledó, E. et al. Abyssal Pacific Seafloor Megafauna Atlas (version 1). *Zenodo*10.5281/zenodo.7765164 (2023).

[56] Horton, T. et al. Recommendations for the standardisation of open taxonomic nomenclature for image-based identifications. *Front. Marine Sci.*10.3389/fmars.2021.620702 (2021).

[57] Ilich, A. R., Misiuk, B., Lecours, V. & Murawski, S. A. MultiscaleDTM: an open-source R package for multiscale geomorphometric analysis. *Trans. GIS* **27**, 1164–1204 (2023).

[58] Highlight seabed images taken by ROV in the Ocean Minerals Company (OMCO) during expedition JC241 in the Clarion–Clipperton Zone (Pacific Ocean, 2023). Published Data Library (National Oceanography Centre, British Oceanographic Data Centre, 2025); 10.5285/2e5a5010-4abd-2beb-e063-7086abc0b159.

