# Supplementary material for: Long-term impact and biological recovery in a deep-sea mining track
Source: Nature. 2025 Mar 26;642(8066):112–8. doi: 10.1038/s41586-025-08921-3 (PMC12137123; doi:10.1038/s41586-025-08921-3)
Supplement: Supplementary file 2 — Reporting Summary [file 41586_2025_8921_MOESM2_ESM.pdf]

nature portfolio

Corresponding author(s): Daniel Jones

Last updated by author(s): 3 July 2024

Reporting Summary

Nature Portfolio wishes to improve the reproducibility of the work that we publish. This form provides structure for consistency and transparency in reporting. For further information on Nature Portfolio policies, see our Editorial Policies and the Editorial Policy Checklist.

Statistics

For all statistical analyses, confirm that the following items are present in the figure legend, table legend, main text, or Methods section.

n/a	Confirmed	
☐	☒	The exact sample size (*n*) for each experimental group/condition, given as a discrete number and unit of measurement
☐	☒	A statement on whether measurements were taken from distinct samples or whether the same sample was measured repeatedly
☐	☒	The statistical test(s) used AND whether they are one- or two-sided<br>*Only common tests should be described solely by name; describe more complex techniques in the Methods section.*
☒	☐	A description of all covariates tested
☐	☒	A description of any assumptions or corrections, such as tests of normality and adjustment for multiple comparisons
☐	☒	A full description of the statistical parameters including central tendency (e.g. means) or other basic estimates (e.g. regression coefficient) AND variation (e.g. standard deviation) or associated estimates of uncertainty (e.g. confidence intervals)
☐	☒	For null hypothesis testing, the test statistic (e.g. F, t, r) with confidence intervals, effect sizes, degrees of freedom and P value noted<br>*Give P values as exact values whenever suitable.*
☒	☐	For Bayesian analysis, information on the choice of priors and Markov chain Monte Carlo settings
☒	☐	For hierarchical and complex designs, identification of the appropriate level for tests and full reporting of outcomes
☒	☐	Estimates of effect sizes (e.g. Cohen's *d*, Pearson's *r*), indicating how they were calculated

Our web collection on statistics for biologists contains articles on many of the points above.

Software and code

Policy information about availability of computer code

Data collection	Data collecting and handling were implemented using standard methods, software tools and functions detailed in the Methods.
Data analysis	Data analyses were implemented using standard methods, software tools and functions detailed in the Methods.

For manuscripts utilizing custom algorithms or software that are central to the research but not yet described in published literature, software must be made available to editors and reviewers. We strongly encourage code deposition in a community repository (e.g. GitHub). See the Nature Portfolio guidelines for submitting code & software for further information.

Data

Policy information about availability of data

All manuscripts must include a data availability statement. This statement should provide the following information, where applicable:

- Accession codes, unique identifiers, or web links for publicly available datasets
- A description of any restrictions on data availability
- For clinical datasets or third party data, please ensure that the statement adheres to our policy

Data generated for this study are available in the supplementary material. The images of the seafloor used for analysis are available at https://doi.org/10.5285/2392b266-126b-db3f-e063-7086abc0fe00 (images taken in 2023) and https://doi.org/10.5285/27e550f2-81ff-6bf8-e063-7086abc04f4f (images taken in 1970s). The Abyssal Pacific Seafloor Megafauna Atlas (APSMA image-based taxonomical catalogue) used in the identification of organisms in this study is

available at https://doi.org/10.5281/zenodo.7765164 . Data handling and analyses were implemented using standard methods, software tools and functions detailed in the Methods.

Research involving human participants, their data, or biological material

Policy information about studies with human participants or human data. See also policy information about sex, gender (identity/presentation), and sexual orientation and race, ethnicity and racism.

Reporting on sex and gender	N/A
Reporting on race, ethnicity, or other socially relevant groupings	N/A
Population characteristics	N/A
Recruitment	N/A
Ethics oversight	N/A

Note that full information on the approval of the study protocol must also be provided in the manuscript.

Field-specific reporting

Please select the one below that is the best fit for your research. If you are not sure, read the appropriate sections before making your selection.

☐ Life sciences ☐ Behavioural & social sciences ☒ Ecological, evolutionary & environmental sciences

For a reference copy of the document with all sections, see nature.com/documents/nr-reporting-summary-flat.pdf

Ecological, evolutionary & environmental sciences study design

All studies must disclose on these points even when the disclosure is negative.

Study description	The study collected quantitative data from a site disturbed by a mining vehicle in 1979, 44 year prior to the expedition. There were up to five treatment factors used: 1) “collection tracks” on the vehicle track, 2) “propulsion tracks” impacted by the vehicle propulsion system, 3) “plume areas” adjacent to the track which were assumed from plume models to be impacted by sedimentation disturbance, 4) a “control” site ~2 km east from the OMCO test area, and 5) “pre-disturbance” obtained prior to the mining vehicle test. The number of sampling units obtained was variable depending on the analysis, but ranged between 3 and 12 for each treatment, except for the carbon uptake measurements (n = 2).
Research sample	For faunal samples, the research samples consisted of all animals within a set size fraction: megafauna (>10mm) and macrofauna (>300 µm). These are commonly used and standard groups used in deep-water studies and recognised by the International Seabed Authority.
Sampling strategy	The size of sampling units was set based on standard practice for macrofauna (a 0.25m2 boxcore; e.g. DOI: 10.3390/jmse4010002) and based on sampling unit size evaluation (e.g. DOI: 10.1016/j.pocean.2018.11.003) for photographic samples of megafauna. Sediment samples were obtained using standard approaches.
Data collection	Data were collected on a seagoing expedition on the Research Vessel James Cook. A range of methods were used for data collection, including remotely operated vehicles and sediment samplers as detailed in the paper. BF, ESL and LVA analysed images and provided megafaunal data with input from BOM, AG and DJ. BA, CB, GBC, LK, ES and AG analysed macrofaunal data. SB, LM and RJ analysed the sediment biogeochemistry data. CW, TLB and VH collected and processed seafloor acoustic data. AS collected and analysed microbial data. LM and SE collected and processed water samples. AD and DA analysed physical oceanography data.
Timing and spatial scale	All data were obtained within 10 km of the mining collector test site centred 13°44’N 126°13.5’W. Samples, imagery and other data were collected during RRS James Cook expedition JC241 between 14 February and 12 March 2023. Photographs were also obtained in the area of the collector test during three cruises of the RV Governor Ray 31. Two cruises were carried out before the test (June 1978: GR7801; November 1978: GR7804) and one after (October 1979: GR7904).
Data exclusions	No data were excluded from analysis.
Reproducibility	The original dataset generated and used to run the analyses is provided as supplementary material, the software and standard code functions used are all open-sourced, the images used for analysis are publically-accessible and the taxonomic identification catalogue used to standardize specimen identifications is available on-line.
Randomization	Physical samples were obtained at randomised positions within each treatment area. Photographic data were collected over wider areas and randomly subsampled to create sampling units used for analysis.
Blinding	We used BIIGLE 2.0 to blind the location of the images containing each of the specimen occurrences during image re-analysis to

Blinding	minimize potential observer-bias. Several spatially-blind reviews were conducted to the whole dataset to ensure a robust taxonomic alignment between data from different sites, consisting in side-by side visualization of all specimens classified under the same catalogue label using the 'Label Review Grid Overview' tool in BIIGLE 2.0.

Did the study involve field work? ☒ Yes ☐ No

Field work, collection and transport

Field conditions	The near-seabed oceanographic conditions are similar between the OMCO test and control areas, with typical Clarion Clipperton Zone (CCZ) seabed temperature (1.48°C in situ), oxygen (151 µmol/kg) and absolute salinity (34.87 g kg-1) and current speeds (mean velocity from three lowered ADCP profiles 8 m above the seabed: 40 mm/s, direction 170°). Nodules in the collector area were abundant (from boxcore samples: mean 155 nodules m-2, 18.6 kg nodules m-2) and relatively large (largest dimension: mean 72± 19 mm S.D., maximum observed 152 mm) but within the range of other exploration areas within the CCZ. Most nodules were found at the sediment surface. Natural sedimentation rates across the CCZ are low (1.5 – 11.5 mm kyr-1).
Location	All data were obtained within 10 km of the collector test site centred 13°44'N 126°13.5'W. Water depth was around 4700 m.
Access & import/export	All samples and data were obtained as part of permitted marine scientific research in areas beyond national jurisdiction (the high seas and seabed beyond the limits of the continental shelf). Appropriate diplomatic clearance was obtained for the expedition transits. Some areas sampled during the expedition were licenced for deep-sea mining exploration, in all cases the relevant contract holder was notified prior to the expedition as was the International Seabed Authority. All CITES species obtained during the expedition were imported into the UK under licence no 627172/01 (Antipatharia) and 627172/02 (Scleractinia) issued by the Animal and Plant Health Agency, UK on 13 January 2023.
Disturbance	Disturbance caused by the study was minimal, with most operations being done using remote sensing. We followed the UK NERC's Marine Environment Interaction Policy (MEIP). The impact of acoustic data collection was assessed using a NERC Marine Environmental Mitigation Plan, which included marine mammal observations by trained marine mammal observers prior to start of data acquisition and soft-start procedures to mitigate potential impacts.

Reporting for specific materials, systems and methods

We require information from authors about some types of materials, experimental systems and methods used in many studies. Here, indicate whether each material, system or method listed is relevant to your study. If you are not sure if a list item applies to your research, read the appropriate section before selecting a response.

Materials & experimental systems

n/a	Involved in the study	
☒	☐	Antibodies
☒	☐	Eukaryotic cell lines
☒	☐	Palaeontology and archaeology
☐	☒	Animals and other organisms
☒	☐	Clinical data
☒	☐	Dual use research of concern
☒	☐	Plants

Methods

n/a	Involved in the study	
☒	☐	ChIP-seq
☒	☐	Flow cytometry
☒	☐	MRI-based neuroimaging

Animals and other research organisms

Policy information about studies involving animals; ARRIVE guidelines recommended for reporting animal research, and Sex and Gender in Research

Laboratory animals	No laboratory animals were used in this study
Wild animals	The study included the capture of invertebrate organisms (no cephalopods) in the field. Animals were recovered by remotely operated vehicle or collected with sediment or water samples. Organisms recovered to the ship are killed by the temperature and pressure change between the seafloor and the surface. Most organisms obtained for this study were small < 10 mm and included polychaetes, arthropods, bryozoans, echinoderms, molluscs, priapulids and nemerteans. The species, strains and ages of these animals are not known.
Reporting on sex	N/A
Field-collected samples	No laboratory work on field-collected samples was carried out for this study
Ethics oversight	No ethical approval or guidance

Note that full information on the approval of the study protocol must also be provided in the manuscript.

Plants

Seed stocks	N/A
Novel plant genotypes	N/A
Authentication	N/A
